# Analysis of Retinal Layer Thicknesses and Their Clinical Correlation in Patients with Traumatic Optic Neuropathy

**DOI:** 10.1371/journal.pone.0157388

**Published:** 2016-06-13

**Authors:** Ju-Yeun Lee, Kyuyeon Cho, Kyung-Ah Park, Sei Yeul Oh

**Affiliations:** Department of Ophthalmology, Samsung Medical Center, Sungkyunkwan University School of Medicine, Seoul, Korea; University of Iowa, UNITED STATES

## Abstract

The aims of this study were 1) To evaluate retinal nerve fiber layer (fRNFL) thickness and ganglion cell layer plus inner plexiform layer (GCIPL) thickness at the fovea in eyes affected with traumatic optic neuropathy (TON) compared with contralateral normal eyes, 2) to further evaluate these thicknesses within 3 weeks following trauma (defined as “early TON”), and 3) to investigate the relationship between these retinal layer thicknesses and visual function in TON eyes. Twenty-nine patients with unilateral TON were included. Horizontal and vertical spectral-domain optical coherence tomography (SD-OCT) scans of the fovea were taken in patients with unilateral TON. The main outcome measure was thickness of the entire retina, fRNFL, and GCIPL in eight areas. Thickness of each retinal layer was compared between affected and unaffected eyes. The correlation between the thickness of each retinal layer and visual function parameters, including best corrected visual acuity, color vision, P100 latency, and P100 amplitude in visual evoked potential (VEP), mean deviation (MD) and visual field index (VFI) in Humphrey visual field analysis in TON eyes was analyzed. Thicknesses of the entire retina, fRNFL, and GCIPL in SD-OCT were significantly thinner (3–36%) in all measurement areas of TON eyes compared to those in healthy eyes (all *p*<0.05). Whereas, only GCIPL in the outer nasal, superior, and inferior areas was significantly thinner (5–10%) in the early TON eyes than that in the control eyes (all *p*<0.01). A significant correlation was detected between retinal layer thicknesses and visual function parameters including color vision, P100 latency and P100 amplitude in VEP, MD, and VFI (particularly P100 latency, MD, and VFI) (r = -0.70 to 0.84). Among the retinal layers analyzed in this study, GCIPL (particularly in the superior and inferior areas) was most correlated with these five visual function parameters (r = -0.70 to 0.71). Therefore, evaluation of morphological change of each retinal layer using SD-OCT can help in understanding TON pathophysiology and indirectly assessing visual function. Moreover, evaluation of the morphological change of the GCIPL in TON eyes may be useful to assess visual function in patients with early TON.

## Introduction

Previous studies have investigated the functional and morphological changes in the retina following optic nerve injury in patients with various optic nerve disorders, such as glaucoma, ischemic optic neuropathy, and optic neuritis.[1–4] Optic nerve injury in animal models develops axonal damage and retinal ganglion cell (RGC) degeneration.[1, 3] These degenerative changes lead to morphological changes in the retina and significant thinning of retinal layers.

Spectral-domain optical coherence tomography (SD-OCT) has been widely adopted as noninvasive method to track structural changes in retinal layers. SD-OCT can delineate structures with an axial resolution of 5–7 μm,[5] and provides clear imaging of retinal layers to help diagnose optic nerve disorders. SD-OCT cross-sectional images provide quantitative morphological information of retinal layers. Thus, SD-OCT has been used to measure retinal thickness for evaluating structural damages in the peripapillary area and macula in patients with optic nerve disorders.

Peripapillary RNFL (cpRNFL) thickness measured by SD-OCT could be a quantitative marker of optic nerve disorders.[6] Along with cpRNFL, loss of the macular ganglion cell complex reflects structural damage to RGCs and their axons following optic nerve damage.[7] The macular ganglion cell complex consists of three retinal layers, including the nerve fiber layer (NFL), the ganglion cell layer (GCL), and the inner plexiform layer (IPL) and can be measured by SD-OCT; thus, many trials have evaluated the macular ganglion cell complex using SD-OCT in patients with many optic nerve disorders.[8–12]

Traumatic optic neuropathy (TON) refers to optic nerve injury secondary to trauma and causes acute axonal loss with severe visual impairment. The mechanism underlying axonal injury-induced RGC degeneration can also occur in patients with TON. A number of pre-clinic studies have demonstrated retinal neural degeneration in animal models of TON using SD-OCT.[1, 13, 14] They reported early thinning of the cpRNFL and early loss of RGC density after optic nerve injury.[13]

Although many studies have demonstrated morphological or histological changes in the retina of animal models following optic nerve injury,[1, 13–17] few studies have clinically assessed morphological changes in retinal layer thickness at the fovea using SD-OCT in patients with early stage TON. In addition, no study has evaluated the relationship between thicknesses of the retinal layers and visual function in patients with TON.

Therefore, the aims of this study were 1) to evaluate the RNFL and GCL + IPL (GCIPL) thicknesses in four different directions of the fovea in TON eyes compared with those in contralateral normal eyes, 2) to further evaluate these thicknesses within 3 weeks after trauma (early stage TON), and 3) to investigate the relationship between thicknesses of these retinal layers and visual function in TON eyes.

## Methods

This hospital-based retrospective observational study was a single center study performed from 2012 to 2014 in accordance with the tenets of the Declaration of Helsinki. This study was approved by the institutional review board of the Samsung Medical Center. Patient records were anonymized and de-identified prior to analysis.

All subjects had been diagnosed with unilateral indirect TON and had undergone SD-OCT (Heidelberg Engineering, Vista, CA, USA) within 3 months after trauma. Subjects with various factors that could affect OCT measurements and visual clarity except TON, such as retinal disease, vitreous hemorrhage, glaucoma, or other ocular conditions associated with trauma, including angle recession and choroidal rupture, or a history of ocular surgery, or other intracranial lesions were excluded. Patients who underwent SD-OCT more than 3 months after trauma were also excluded.

Unilateral indirect TON was diagnosed by a history of blunt trauma, clinical manifestations with the evidence of optic neuropathy, and magnetic resonance imaging (MRI) of the orbit. TON was characterized by subconjunctival hemorrhage, decreased visual acuity and color vision with a relative afferent pupillary defect in one eye, a normal fundus, and no immediate changes in the optic nerve with MRI findings consistent with optic neuropathy during the early post-traumatic period in the context of a history of non-penetrating trauma to the forehead or malar region. Since indirect TON is a clinical diagnosis typically made when there is evidence of optic neuropathy related to blunt trauma,[18] patients who had a history of blunt trauma and clinical manifestations with the evidence of optic neuropathy but normal MRI findings, were also considered to have TON, and they were enrolled in this study.

T1 and T2 weighted, fat suppressed MRI images were acquired axially through the orbit and some parts of the brain including the intracranial portion of the optic nerve. The images which showed hyper-intensity in the unilateral optic nerve or optic nerve swelling without the presence of optic canal fracture, a displaced bony fragment impinging upon the optic nerve, a metallic foreign body in the orbit, or an optic nerve sheath hematoma were considered to indicate ‘TON’.

All study participants underwent a detailed ophthalmologic examination, including assessment of refraction, best-corrected visual acuity (BCVA), color vision, relative afferent pupillary defect, slit-lamp examination of the anterior segment, tonometry, and a fundus examination. All patients underwent MRI of the orbit, pattern visual evoked potential (VEP) testing, automated perimetry using the central 30–2 VF test (HFA model 460; Humphrey Instruments Inc., San Leandro, CA, USA) and macular and disc SD-OCT at the first visit. Data on sex, age, BCVA, color vision, P100 latency and P100 wave amplitude of the VEP, the mean deviation (MD) value, and visual field index (VFI) on the Humphrey visual field analysis were collected from the electronic records of each patient. Corrected visual acuities were transformed to a logarithmic scale (logMAR) for statistical analysis.

Color vision data were collected using Ishihara color vision test 24 plate edition. The test was performed in a well-lit room with a light level of approximately 325 lux, and no glare on the patient’s screen. The plates were held 75cm from the subject and tilted so that the plane of the paper was at right angles to the line of vision. The numerals which were seen on plates were stated, and each answer should be given without more than three seconds delay. In this study, since the color vision test was designed to separate the color defectives from those with normal color appreciation, color vision test was performed with 15 plates. Patients who have congenital color vision deficiency were excluded. Color vision was recorded as the number of correct answers in a set of 15 plates.[19]

PVEPs were recorded by Nihon Kohden MEB-2200 Neuropack (Tokyo, Japan). In accordance with the recommendations of the International Society for Clinical Electrophysiology of Vision,[20] active electrodes were placed on the scalp over the visual cortex at Oz, with the reference electrode at Fz. A separate electrode was attached at vertex Cz and connected to the ground. The visual stimulus was a black and white check board pattern generated on a television monitor. The check size was 20 minutes of arc. The contrast was 100%, and the mean luminance was kept at 50 cd/m^2^. The stimulus field of the pattern was 16×16 degrees. The pattern was reversed at three reversals per second for transient VEP, and 10 reversals per second for steady-state VEP. The electrodes were connected to a preamplifier with a bandpass of 1 to 100 Hz, and for each measurement, 100 responses were averaged. The patient fixed on a point in the center of the pattern monocularly from an observing distance of 70 cm, with an undilated pupil under full refractive correction. VEP was performed in each eye of the same patient with simultaneous stimulus and same method regardless of a presence of TON. To verify the data quality, we also performed VEP with binocular stimulation (both eyes simultaneously) as well as monocular stimulation. For example, when a normal response was reported in both eyes whereas a delayed response of the P100 wave was reported in the right eye, VEP was performed again for obtaining accurate results based on the possibility of a false response in the right eye. Fixation cooperation was monitored closely by an experienced technician. For accurate measurements during the PVEP test, all patients were evaluated by one examiner. Data on latency and amplitude of P100 wave were collected for analysis in this study.

VFI was introduced for estimating the rate of change in visual field on Humphrey field analysis. This index addresses several shortcomings of the MD, and unlike the MD, the VFI expresses the amount of visual field loss as a percentage compared to the sensitivity of a healthy reference group. A VFI of 100% would be observed in a completely normal visual field, whereas a VFI of 0% in a perimetrically blind visual field.[21] Since MD and VFI have a significant correlation and can provide equivalent information about the visual field,[22, 23] we used both parameters for analysis in this study.

All OCT scans were performed with a SD-OCT that provided 40,000 A-scans/sec with 7 μm optical and 3.5 μm digital axial resolution. Horizontal and vertical SD-OCT scans of the fovea were obtained in all subjects. All OCT images were converted to grey scale for better visualization and accurate analysis. The thickness of each retinal layer was measured manually using in the SD-OCT caliper instrument.

**Fig 1:**
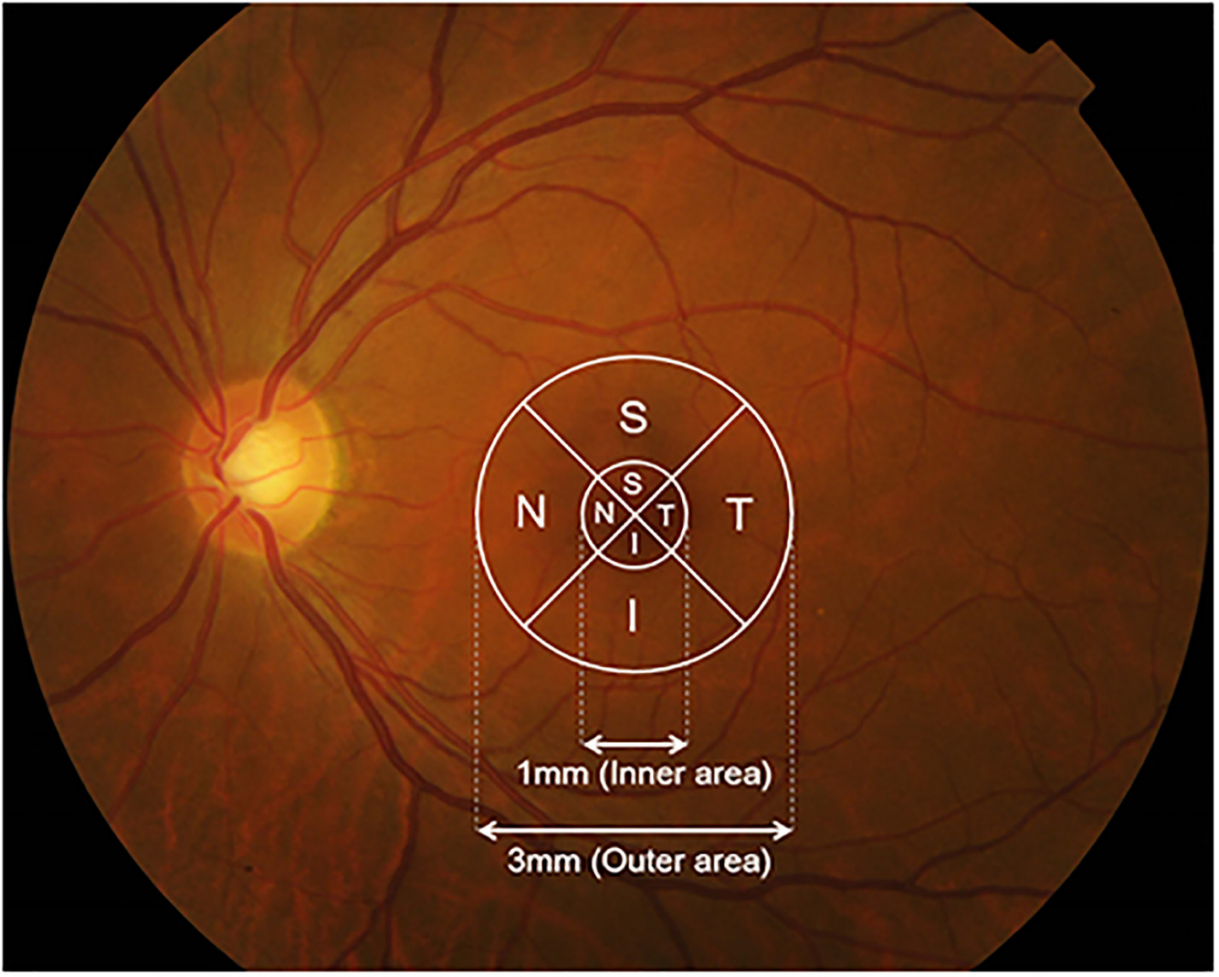
Thickness data from the map of each layer were grounded within 2 concentric circles. The retinal areas are displayed on a fundus photograph.

Image measurements were similar to published methods.[24] Thickness at the foveal center was defined as the mean value from horizontal and vertical scans. The thickness values for the inner areas, including the inner superior, inner nasal, inner temporal, and inner inferior areas were measured in the ETDRS central circular 1000μm-diameter area (500μm in either direction from the foveal center) (Fig 1). Thicknesses of the outer areas, including the outer superior, outer nasal, outer temporal, and outer inferior areas were measured in the ETDRS circular 3000μm-diameter area (1500μm in either direction from the foveal center). The only modification compared to the standard ETDRS was that we measured thickness values in four directions even in the smallest circular 1000μm-diameter area.

**Fig 2:**
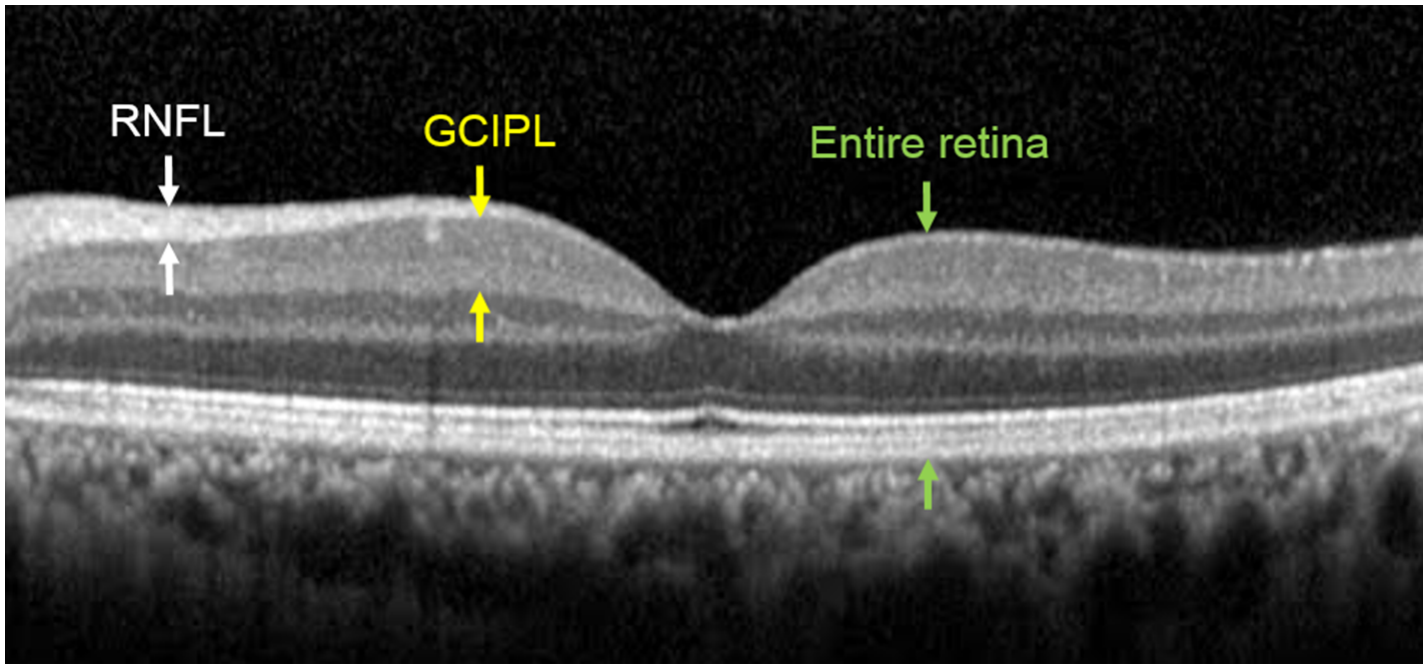
A representative spectral domain optical coherence tomography image obtained in the left eye of one subject in this study. (RNFL, retinal nerve fiber layer; GCIPL, ganglion cell and inner plexiform layer).

Based on this method, thicknesses of the RNFL (fRNFL), GCIPL, and entire retina in the outer areas and thicknesses of the GCIPL and entire retina in the inner areas were measured manually (Fig 2). Since occasionally the automatic boundary line in SD-OCT software did not exactly fit the real boundary of the retinal layer, we measured each thickness value manually with the SD-OCT instrument for a more accurate analysis.

The thicknesses of each retinal layer in affected eyes were compared with measurements at corresponding points in contralateral unaffected eyes. The images were taken by an experienced technician. Two experienced examiners (J.L and K.C), who were blinded to the study results, analyzed all images.

Inter-observer reproducibility was evaluated based on the intraclass correlation coefficient (ICC). The two examiners evaluated the images again, and the same images were used for this evaluation. The intra-observer ICC value was also calculated after this analysis.

Statistical analyses were performed using SAS ver. 9.1.3 software (SAS Institute, Cary, NC, USA). Thickness of each retinal layer was compared using the Wilcoxon signed-rank test, and Spearman’s correlation analysis was used to assess the associations between retinal layers and visual function parameters, including BCVA, color vision, P100 wave latency and amplitude of VEP, MD, and VFI on the Humphrey field analysis. Data within 3 weeks after trauma were considered “early TON” and were analyzed separately. Inter-observer variability and intra-observer repeatability were analyzed using the ICC. A *p-*value < 0.05 was considered significant.

## Results

A total of 29 patients were enrolled (23 males and 6 females; mean age, 42 ± 22 years; range, 8–81 years). All patients had a history of blunt trauma and reported visual impairment just after trauma. The mean time between trauma and the first SD-OCT scan was 5.6 ± 4.0 weeks (40 ± 30 days). Among all subjects, 10 were classified as early TON within 3 weeks after trauma, and their mean time between trauma and the first SD-OCT scan was 1.5 ± 1.0 weeks (12 ± 7 days). Intraocular pressure was within the normal range in every case. No patient had a history of surgery due to a mid-facial or orbital fracture or optic neuropathy. The patient demographics are shown in Table 1. Table 2 presents the descriptive statistics of functional and vision testing, including LogMAR visual acuity, Ishihara color vision test, the Humphrey visual field analysis, and PVEP. Significant differences were observed between affected eyes and unaffected eyes for nine parameters.

**Table 1:** Demographics of patients with unilateral traumatic optic neuropathy.

Variable	Affected eyes (n = 29)	Unaffected eyes (n = 29)	p-value*
	Mean ±SD	Mean ±SD	
Age (years)	42±22		-
Gender (male/female)	23/6		-
Interval between trauma and OCT (days)	40±30 (range, 3–90)		-
Refractive error (prism diopter)	-1.69±2.26	-1.33±1.51	0.25
Intraocular pressure (mmHg)	16.1±3.5	14.5±3.0	0.59

* paired t test

**Table 2:** Descriptive statistics of the functional and vision tests in all patients.

Variable	Affected eyes (n = 29)	Unaffected eyes (n = 29)	p-value*
	Mean ±SD	Mean ±SD	
Visual acuity (LogMAR)	1.79±1.36	0.01±0.06	**<0.01**
Color vision	3.6/15	14.9/15	**<0.01**
(number of correct answers /number of a set of plates)			
Visual field			
Mean deviation (dB)	-17.6±12.6	-1.9±1.4	**<0.01**
Visual field index (%)	50.2±41.1	98.9±9.8	**<0.01**
Visual evoke potential			
Latency of P100 wave (ms)	128.7±31.0	103.2±14.5	**0.04**
Amplitude of P100 wave (μV)	5.9±7.1	8.1±6.0	**0.01**

* *p* < 0.05, Wilcoxon sign-rank test

### Comparison of retinal layer thicknesses at the foveal center in patients with TON

**Fig 3:**
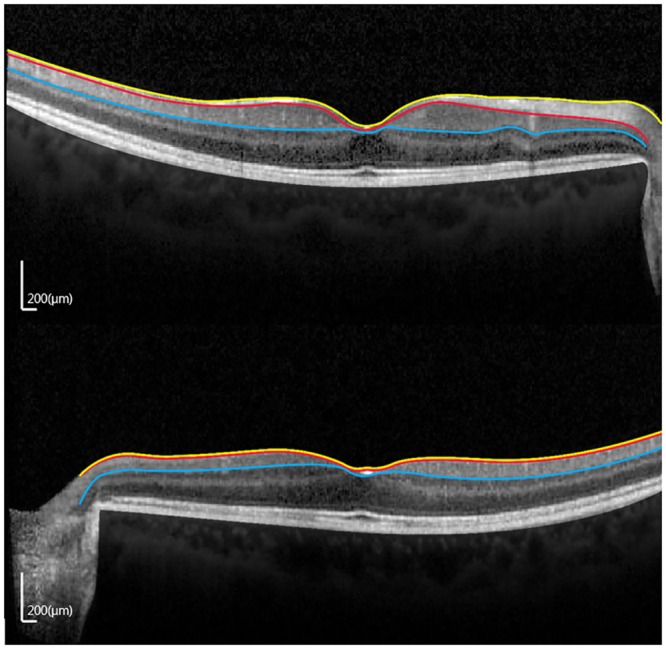
Optical coherence tomography scan images (1:1 pixel views) showing the representative images in eyes with unilateral traumatic optic neuropathy (TON). (A) Contralateral normal eye. (B) TON eye. (RNFL, retinal nerve fiber layer; GCIPL, ganglion cell layer and inner plexiform layer.).

Thicknesses of the entire retina, fRNFL, and GCIPL in patients with all stages of TON are presented in Table 3. All measurements decreased significantly (Fig 3).

**Table 3:** Retinal layer thicknesses measured by spectral domain optical coherence tomography in both eyes of all patients with unilateral traumatic optic neuropathy. All measurements were significantly smaller in TON eyes than those in unaffected eyes. (Wilcoxon sign rank test).

Variable	Affected eyes (n = 29)	Unaffected eyes (n = 29)	p-value*
	Mean ±SD (%)^a^	Mean ±SD	
***Thickness* (μ*m*)**			
**cpRNFL**			
Temporal	59±25 (23%)	77±14	<0.01*
Nasal	51±26 (20%)	64±15	<0.01*
Superior	101±35 (22%)	129±17	<0.01*
Inferior	98±37 (21%)	124±16	<0.01*
**Entire retina**			
*Inner locations*			
Inner temporal	278±10 (3%)	289±22	<0.01*
Inner nasal	286±20 (3%)	296±26	<0.01*
Inner superior	290±18 (3%)	299±21	<0.01*
Inner inferior	291±17 (3%)	298±21	0.03*
*Outer locations*			
Outer temporal	319±22 (4%)	333±15	<0.01*
Outer nasal	335±30 (6%)	358±22	<0.01*
Outer superior	336±28 (6%)	356±17	<0.01*
Outer inferior	327±29 (5%)	344±18	<0.01*
**fRNFL**			
*Outer locations*			
Outer temporal	14±6 (12%)	16±3	<0.01*
Outer nasal	20±10 (29%)	28±5	<0.01*
Outer superior	27±14 (32%)	40±5	<0.01*
Outer inferior	27±12 (36%)	42±6	<0.01*
**GCIPL**			
*Inner locations*			
Inner temporal	33±12 (25%)	44±14	<0.01*
Inner nasal	35±12 (26%)	47±13	<0.01*
Inner superior	37±16 (26%)	50±12	<0.01*
Inner inferior	38±15 (25%)	51±12	<0.01*
*Outer locations*			<0.01*
Outer temporal	72±21 (20%)	90±9	<0.01*
Outer nasal	74±23 (24%)	97±13	<0.01*
Outer superior	71±19 (20%)	89±9	<0.01*
Outer inferior	73±17 (19%)	90±9	<0.01*

SD, standard deviation; cpRNFL, peripapillary retinal nerve fiber layer; fRNFL, foveal retinal nerve fiber layer; GCIPL, ganglion cell layer and inner plexiform layer

^a^ % change of the affected eye compared with the contralateral eye

* *p* < 0.05, Wilcoxon sign-rank test

### Comparison of retinal layer thickness at the foveal center in patients with early TON

**Fig 4:**
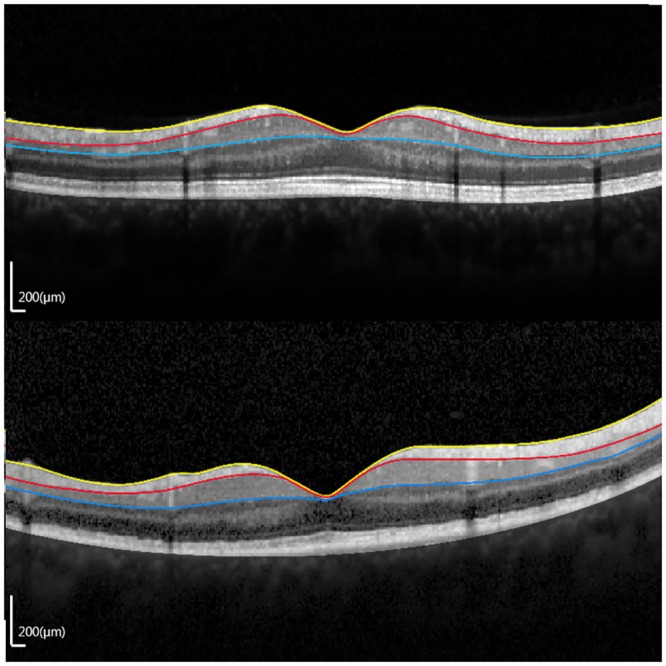
Vertical optical coherence tomography scan images (1:1 pixel views) showing a representative image in eyes with traumatic optic neuropathy (TON) within 3 weeks after trauma (early TON). (A) TON eye. (B) Contralateral unaffected eye. (RNFL, retinal nerve fiber layer; GCIPL, ganglion cell layer and inner plexiform layer.)

Thicknesses of the entire retina, fRNFL, and GCIPL in patients with early TON are presented in Table 4. GCIPL thickness measurements at the outer nasal, superior, and outer inferior areas showed significant reductions (all *p =* 0.005) (Fig 4). No marked reduction in cpRNFL, entire retina, or fRNFL thicknesses was observed in any area.

**Table 4:** Retinal layer thicknesses measured by spectral domain optical coherence tomography in both of patients with early traumatic optic neuropathy (within 3 weeks after trauma). Outer superior and outer inferior GCIPL thicknesses were significantly thinner in the affected eyes than those in unaffected eyes (Wilcoxon sign-rank test).

Variable	Affected eyes (n = 10)	Unaffected eyes (n = 10)	p-value*
	Mean ±SD (%)^a^	Mean ±SD	
***Thickness* (μ*m*)**			
**cpRNFL**			
Temporal	76±26	76±7	0.81
Nasal	71±28	61±12	0.95
Superior	127±29	130±14	0.61
Inferior	124±24	124±15	0.95
**Entire retina**			
*Inner locations*			
Inner temporal	282±18	287±27	0.20
Inner nasal	295±9	293±29	0.99
Inner superior	296±20	299±25	0.45
Inner inferior	299±14	299±27	0.72
*Outer locations*			
Outer temporal	332±14	331±15	0.29
Outer nasal	353±25	358±20	0.77
Outer superior	352±21	353±14	0.96
Outer inferior	344±26	346±14	0.84
**RNFL**			
*Outer locations*			
Outer temporal	17±5	16±2	1.00
Outer nasal	27±8	29±4	0.72
Outer superior	39±10	41±7	0.44
Outer inferior	36±11	41±6	0.11
**GCIPL**			
*Inner locations*			
Inner temporal	38±11	41±13	0.45
Inner nasal	45±9	44±13	0.61
Inner superior	46±10	51±12	0.33
Inner inferior	49±10	51±10	0.58
*Outer locations*			
Outer temporal	86±16	88±11	0.72
Outer nasal	90±18 (5%)	95±10	<0.01*
Outer superior	80±14 (9%)	88±7	<0.01*
Outer inferior	81±11 (10%)	90±4	<0.01*

SD, standard deviation; cpRNFL, peripapillary retinal nerve fiber layer; fRNFL, foveal retinal nerve fiber layer; GCIPL, ganglion cell layer and inner plexiform layer

^a^ % change of the affected eye compared with the contralateral eye

* *p* < 0.05, Wilcoxon sign-rank test

### Correlation between retinal layer thickness and visual function

The correlations between the retinal layer thickness measurements and visual function are presented in Table 5. The MD and VFI on the Humphrey field analysis were significantly correlated with entire retina, fRNFL, and GCIPL thicknesses. P100 latencies were significantly negatively correlated with outer temporal and outer superior fRNFL thicknesses and all GCIPL thickness measurements. Peak to peak P100 amplitude was significantly positively correlated with the GCIPL thickness measurements at all areas, except the outer temporal and outer nasal areas. In addition, color vision was significantly positively correlated with the inner nasal and inner superior GCIPL thickness measurements. LogMAR BCVA was not associated with the retinal thickness measurements.

**Table 5:** Correlation between retinal layer thickness measurements and visual function.

`Variable	TON eyes					
	BCVA	CV	P100(A)	P100(L)	MD	VFI
**Entire retina**						
*Inner locations*						
Inner temporal	-0.176	0.244	-0.050	-0.161*	0.659*	0.615*
Inner nasal	-0.133	0.427	-0.032	-0.178	0.549	0.535
Inner superior	-0.233	0.543	0.195	-0.366*	0.619*	0.634*
Inner inferior	-0.255	0.568	0.179	-0.187*	0.775*	0.761*
*Outer locations*						
Outer temporal	-0.242	0.309	0.295	-0.676*	0.792*	0.838*
Outer nasal	-0.379	0.556	0.307	-0.455*	0.637*	0.717*
Outer superior	-0.241	0.359	0.511	-0.484*	0.709*	0.756*
Outer inferior	-0.420	0.548	0.318	-0.431*	0.659*	0.739*
**fRNFL**						
*Outer locations*						
Outer temporal	-0.276	0.169	0.070	-0.548*	0.336	0.400
Outer nasal	-0.325	0.418	0.655*	-0.494	0.703*	0.657*
Outer superior	-0.399	0.544	0.454	-0.589*	0.675*	0.642*
Outer inferior	-0.509	0.447	0.429	-0.619	0.616*	0.674*
**GCIPL**						
*Inner locations*						
Inner temporal	-0.248	0.479	0.543*	-0.593*	0.306	0.308
Inner nasal	-0.291	0.650*	0.587*	-0.666*	0.558*	0.593*
Inner superior	-0.291	0.632*	0.707*	-0.649*	0.582*	0.577*
Inner inferior	-0.193	0.291	0.689*	-0.548*	0.648*	0.604*
*Outer locations*						
Outer temporal	-0.337	0.403	0.506	-0.683*	0.575*	0.631*
Outer nasal	-0.303	0.315	0.418	-0.700*	0.621*	0.690*
Outer superior	-0.217	0.333	0.554*	-0.550*	0.659*	0.657*
Outer inferior	-0.273	0.481	0.614*	-0.567*	0.501*	0.547*

BCVA, best corrected visual acuity; CV, color vision; P100(A), peak to peak amplitude of P100 wave in visual evoked potential; P100(L), latency of P100 wave in visual evoked potential; MD, mean deviation in Humphrey field analysis; VFI, visual field index in Humphrey field analysis; fRNFL, foveal retinal nerve fiber layer; GCIPL, ganglion cell layer and inner plexiform layer

* *p*<0.05, spearman correlation

### Correlation between retinal thickness and the time after injury

**Fig 5:**
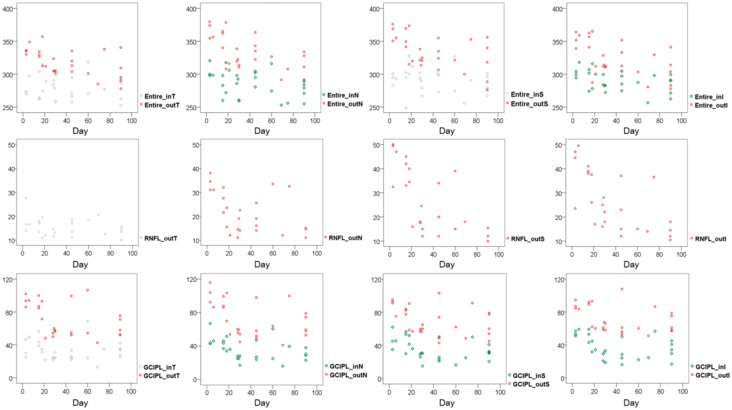
Multi-panel figures showing correlations between retinal thickness and the time after injury using Spearman correlation. (red and green: *p*<0.05, gray: not significant).

The correlations between retinal thickness measurements and the time after injury are presented in Fig 5. All retinal thickness measurements except for outer temporal areas of the entire retina, fRNFL and GCIPL and inner superior area of entire retina were negatively correlated with the time of injury.

Intraobserver reproducibility and interobserver variability was all over 0.80. Excellent agreement was determined for intra- and inter-observer ICC reproducibility for all retinal layer thickness measurements.

## Discussion

Several studies have reported retinal layer thickness to investigate reduced retinal activity following optic nerve injury. Kanamori et al. reported a longitudinal change in thicknesses of the entire retina, cpRNFL, and RGC complex at 2,3,4,12 and 20 weeks after trauma in four patients. Cunha et al. also investigated progressive macular and cpRNFL thickness reduction over the first 12 weeks following traumatic optic neuropathy in three patients.[25] However, most studies had small sample sizes and did not evaluate the relationship between morphological changes in the retina and visual function in patients with TON. Furthermore, most studies estimated cpRNFL to evaluate axonal loss and few studies have focused on foveal RNFL in patients with TON. Therefore, we conducted this study with a larger sample size and evaluated retinal layer thicknesses at the fovea, including the entire retina, fRNFL, and GCIPL using SD-OCT and the relationship between these retinal layer thicknesses and clinical parameters in patients with unilateral TON.

We demonstrated significant thinning of RNFL, GCIPL, and total macular thicknesses at the fovea in TON eyes. As TON results in loss of RGCs and their axons, damage likely affected the RNFL and GCL. In addition, RGC synapses are located in the IPL; thus, changes in this layer are also expected following an optic nerve injury. Hence, we evaluated GCL plus IPL (GCIPL) thickness to assess all possible changes in TON. As GCIPL accounts for up to 40% of total retinal thickness, total retinal thickness can also decrease along with a reduction in GCIPL thickness.[25] These findings agree well with previous studies. A number of authors documented morphological changes in retinal layers by SD-OCT following indirect or direct optic nerve injury which could lead to optic neuropathy.[1, 13, 14, 26] Liu et al. demonstrated a strong correlation between RGC density and retinal layer thickness, and reported an exponential decline in the number of RGCs and significant thinning of corresponding retinal layers on SD-OCT following optic nerve crush in mice.[1] These morphologic changes detected by SD-OCT have also been reported in humans. Kanamori et al. reported that cpRNFL and GCL thicknesses are stable within 1 week after trauma but start to decrease within 2 weeks.[27] Cunha et al. reported a 12% reduction in total macular thickness over 5 weeks in patients with TON.

Interestingly, the timing of the morphological changes in the retina needs special mention with regard to the timing of optic atrophy. Optic disc becomes progressively pale and atrophic 3–5 weeks after trauma.[18] At the timing of OCT, we did not detect any significant change in optic nerve head on the fundus examination or on disc SD-OCT. We detected a tendency for mean fRNFL thickness to decrease, whereas there was a marked reduction in GCIPL thickness at the outer nasal, superior, and inferior locations. This result suggests that the morphological changes in GCIPL may precede the changes in fRNFL in early TON and that it may occur before optic atrophy. This neurodegenerative progression observed early TON is supported by early loss of RGC soma.[1, 3] Munguba et al. reported that RGC soma counts initially decline faster than NFL thickness *in vivo* as an overall measurable change following optic nerve injury in animal model.[13] The histology data showed a >80% reduction in surviving RGCs 3 weeks following optic nerve injury. Mouse RNFL appeared unchanged until ≥70% RGC loss occurred.[26] In addition, fRNFL thickness on SD-OCT does not decreased exponentially in patients with early TON.[28] Thus, these findings support the acute nature of optic nerve injury in which morphological changes derived from early RGC cell body and dendrite loss precede those from axonal loss and optic nerve atrophy.

Furthermore, our results show segmented RGC loss according to location after trauma. A 5–10% reduction in GCIPL thickness was observed at the outer nasal, superior, and inferior areas in patients with early TON, and thinning of the GCIPL was more distinct in the outer superior and inferior areas than that in the outer nasal area. This pattern of the early GCIPL damage is similar to glaucomatous damage in which relatively large reduction of GCIPL in the superior and inferior area, whereas sparing the GCIPL in the temporal area. In glaucoma, arcuate fibers passing through the superior and inferior portions of the laminar cribrosa are generally known as the most vulnerable zone due to less connective tissue support, whereas the temporal portion is the last to be damaged.[29] In TON, at the moment of trauma, an instantaneous external force can cause injury in the lamina cribrosa particularly the vulnerable superior and inferior portions, and this can lead to axonal damage in the injured areas. Although the reason for this result is unclear, it is assumed that mechanisms responsible for death of RGCs in TON may be partially consistent with glaucoma. This neurodegenerative progression provides valuable information regarding early TON pathophysiology.

We also found a significant correlation between structural damage to the retina and visual function change. The results showed that greater thinning of the RNFL and GCIPL was correlated with a visual field defect. We observed a 30% reduction in RNFL thickness and at least a 25% reduction in GCIPL thickness in eyes with TON. One study reported that a reduction of at least 25% in the RGC complex is correlated with statistical abnormalities in automated perimetry.[30] The results of the present study accord well with previous studies that report the correlation between structural damage to the axons of the optic nerve and visual field defect in various optic neuropathies.[31–36] Dotan et al. demonstrated a significant correlation between cpRNFL thickness and MD of the visual field (r = 0.457) in non-arteritic anterior ischemic optic neuropathy.[33] Le and associates also reported a significant regional correlation between GCC loss, RNFL loss, and visual field defects in glaucoma.[34] In addition, Lennartsson and the associates demonstrated that reduced cpRNFL thickness was correlated with MD in patients with immature optic radiation which causes severe RNFL loss.[36] Based on these studies, we can consider fRNFL and GCIPL thickness as useful predictors for visual field defects in patients with TON.

We observed that the mean MD value in the eye unaffected with TON was -1.9 dB in this study. This can be explained by the fact that four patients had cataract which could affect MD in unaffected eyes. These four patients had normal color vision, no optic disc pallor, neither p100 delay nor p100 amplitude decrease, and no abnormal MRI findings. Hence, we considered that these eyes were not affected by TON, and we enrolled them in this study. Despite this condition, the mean MD value was normal within -2.0 dB in eyes unaffected by TON.[37]

We also detected a significant correlation between retinal layer thickness and the PVEP results. VEP has long been used as an objective test of visual function, and it is compatible with RGC function.[38, 39] Prolonged P100 latency is more reliable than reduced P100 wave amplitude to evaluate visual function.[40] In this study, P100 latency was correlated with retinal layer thickness measurements in 17 areas, whereas P100 amplitude was correlated with retinal layer thickness measurements in only seven areas. Among all retinal layers analyzed in this study, the GCIPL was most correlated with the PVEP parameters, and some GCIPL thickness values were strongly correlated with PVEP parameters (>0.7). These findings suggest that we can anticipate visual function based on thicknesses of the retinal layers, especially the GCIPL. However, one study reported that PVEP did not have any significant correlation with RNFL thickness. In this study, results showed that the degree of correlation between PVEP parameters and entire retina and RNFL thickness was not very strong (<0.7). Thus, caution should be exercised while interpreting this data. However, since there is no study that evaluated the correlation between PVEP parameters and GCIPL thickness, further well-controlled study is needed to verify this relatively strong relationship between PVEP and GCIPL.

Notably, GCIPL (particularly at the superior and inferior areas) was the most correlated with visual function parameters among the retinal layers analyzed in this study. It seems that GCIPL was highly associated with visual function in TON. Therefore, evaluating GCIPL damage in TON eyes compared with contralateral normal eyes may be useful to assess visual function.

The following limitations were encountered in this study. First, this was a single-center study and the sample size was relatively small. Second, longitudinal changes in the thickness of each retinal layer after trauma could not be evaluated. We speculate that post-traumatic fRNFL and GCIPL thicknesses may progress to become abnormally thin because of gradual axon degeneration from post-traumatic autophagy. Therefore, further studies detecting longitudinal changes are necessary. Third, color vision data were collected by well-trained ophthalmology residents as part of routine clinical evaluation using Ishihara test which is not perfectly suitable for a precise diagnosis of acquired color vision abnormalities. Hence, although the color vision data were useful for analysis in this study since they were all recorded in the same way, caution should be exercised while comparing this data with other studies.

Because of the retrospective nature of this study, we could not perform electroretinogram (ERG) to evaluate RGC dysfunction in all patients. We expected that thinning of the GCIPL in TON may be correlated with the amplitude decrease in ERG. Moreover, in addition to the parameters including MD and VFI, a more detailed approach that attempts to match retinal thickness in certain locations with retinal light sensitivity in these areas is needed to establish better correlation between structure and function. Finally, there was a potential for some measurement errors because each retinal layer thickness was measured manually, not using software. However, the measurements were done by two masked observers, and good inter-and intra-observer agreement was demonstrated in most areas of measurement.

In conclusion, regardless of these limitations, we found significant thinning of the entire retina, fRNFL, and GCIPL in TON eyes, and a remarkable reduction of GCIPL in early phase TON. We also demonstrated a correlation between morphological changes in the retinal layers and visual functions, including visual field defect and P100 latency and amplitude. Therefore, analyzing each retinal layer using SD-OCT was helpful to understand TON pathophysiology and assess optic nerve function. Moreover, evaluating the GCIPL in TON eyes may be useful to assess morphological damage and visual function change in patients with early TON. Further studies with large sample sizes are required to verify our results.

## Supporting Information

S1 Table Difference in retinal thickness between unaffected eyes and affected eyes in early traumatic optic neuropathy. (DOCX)
