# Supplementary material for: Analysis of Retinal Layer Thicknesses and Their Clinical Correlation in Patients with Traumatic Optic Neuropathy
Source: PLoS One. 2016 Jun 13;11(6):e0157388. doi: 10.1371/journal.pone.0157388 (PMC4905630; doi:10.1371/journal.pone.0157388)
Supplement: S1 Table — (DOCX) [file pone.0157388.s001.docx]

**S1 Table**. Difference in retinal thickness between unaffected eyes and affected eyes in early traumatic optic neuropathy. All values were calculated as ‘(retinal thickness measurements in unaffected eye) – (retinal thickness measurements in affected eye)’

| ` |  | **Entire retina** | | | | | | | | | **fRNFL** | | | | **GCIPL** | | | | | | | |
| --- | --- | --- | --- | --- | --- | --- | --- | --- | --- | --- | --- | --- | --- | --- | --- | --- | --- | --- | --- | --- | --- | --- |
| **No** | **day** | **aveFT** | **inT** | **inN** | **inS** | **inI** | **outT** | **outN** | **outS** | **outI** | **outT** | **outN** | **outS** | **outI** | **inT** | **inN** | **inS** | **inI** | **outT** | **outN** | **outS** | **outI** |
| 1 | 3 | 3 | 13 | 8 | 5 | -11 | 19 | 5 | -1 | -5 | -12 | -7 | -13 | -3 | 3 | 14 | 8 | 2 | 10 | 1 | -2 | 4 |
| 2 | 3 | 5 | 69 | 37 | 20 | -30 | 12 | 5 | -13 | -23 | -15 | -9 | 1 | -2 | -3 | 1 | 5 | -20 | 10 | 9 | -3 | -18 |
| 3 | 4 | -3 | -5 | -4 | -7 | 2 | 2 | -13 | 10 | -2 | 2 | -11 | 5 | -3 | -3 | -8 | -2 | 5 | 15 | -15 | 4 | -3 |
| 4 | 6 | 9 | 17 | 13 | 6 | 15 | 15 | -4 | -13 | 5 | 1 | -2 | 1 | -1 | 5 | 3 | -16 | -3 | -3 | -8 | -20 | 11 |
| 5 | 14 | -10 | -27 | -19 | 12 | -5 | -10 | -11 | -11 | 0 | -7 | -1 | -1 | -5 | 1 | 5 | 5 | -6 | -6 | -8 | -8 | 8 |
| 6 | 15 | -18 | -12 | -15 | -28 | -33 | -7 | -43 | -19 | -15 | -11 | -17 | -5 | 2 | -1 | 2 | -20 | -23 | 2 | 16 | 11 | 4 |
| 7 | 15 | 7 | 13 | 10 | 9 | 2 | 10 | 1 | 5 | 24 | 12 | 15 | 2 | 4 | -3 | 3 | 2 | 12 | -1 | 3 | 4 | 13 |
| 8 | 18 | -11 | -3 | -7 | -1 | 6 | -3 | 12 | -12 | -12 | -16 | -15 | 3 | 1 | -3 | -4 | 12 | -12 | -3 | 12 | 6 | 12 |
| 9 | 19 | 25 | -7 | 9 | 7 | 11 | -21 | 23 | -1 | 16 | 16 | 28 | -6 | 10 | 6 | 9 | 1 | -1 | -2 | 18 | 9 | 7 |
| 10 | 20 | -4 | -6 | -5 | 31 | 24 | 14 | 24 | 37 | 54 | 41 | 37 | 5 | 23 | 17 | 20 | 38 | 36 | 30 | 25 | 53 | 45 |

aveFT,(Foveal horizontal thickness + Foveal vertical thickness)/2, fRNFL; foveal retinal nerve fiber layer; GCIPL, ganglion cell layer and inner plexiform layer
